# Using machine learning to predict the risk of short-term and long-term death in acute kidney injury patients after commencing CRRT

**DOI:** 10.1186/s12882-024-03676-x

**Published:** 2024-07-30

**Authors:** Menglei Gu, Yalan Liu, Hongbin Sun, Haitong Sun, Yufei Fang, Luping Chen, Lu Zhang

**Affiliations:** Affiliated Hospital of Nanjing University of Chinese Medicine, Jiangsu Province Hospital of Chinese Medicine, Nanjing, China

**Keywords:** Machine learning, Acute kidney injury, Risk stratification tool, Mortality, Continuous renal replacement treatment

## Abstract

**Background:**

The mortality rate and prognosis of short-term and long-term acute kidney injury (AKI) patients who undergo continuous renal replacement therapy (CRRT) are different. Setting up risk stratification tools for both short-term and long-term deaths is highly important for clinicians.

**Method:**

A total of 1535 AKI patients receiving CRRT were included in this study, with 1144 from the training set (the Dryad database) and 391 from the validation set (MIMIC IV database). A model for predicting mortality within 10 and 90 days was built using nine different machine learning (ML) algorithms. AUROC, F1-score, accuracy, sensitivity, specificity, precision, and calibration curves were used to assess the predictive performance of various ML models.

**Results:**

A total of 420 (31.1%) deaths occurred within 10 days, and 1080 (68.8%) deaths occurred within 90 days. The random forest (RF) model performed best in both predicting 10-day (AUROC: 0.80, 95% CI: 0.74–0.84; accuracy: 0.72, 95% CI: 0.67–0.76; F1-score: 0.59) and 90-day mortality (AUROC: 0.78, 95% CI: 0.73–0.83; accuracy: 0.73, 95% CI: 0.69–0.78; F1-score: 0.80). The importance of the feature shows that SOFA scores are rated as the most important risk factor for both 10-day and 90-day mortality.

**Conclusion:**

Our study, utilizing multiple machine learning models, estimates the risk of short-term and long-term mortality among AKI patients who commence CRRT. The results demonstrated that the prognostic factors for short-term and long-term mortality are different. The RF model has the best prediction performance and has valuable potential for clinical application.

**Supplementary Information:**

The online version contains supplementary material available at 10.1186/s12882-024-03676-x.

## Introduction

Acute kidney injury (AKI) is a clinical syndrome involving various clinical presentations and is characterized by a rapid deterioration of kidney function, with an occurrence rate as high as 50-60% among critically ill patients in the intensive care unit (ICU) [1–3]. Renal replacement therapy (RRT) is frequently used to mitigate the adverse effects of AKI, especially in patients with severe complications such as hyperkalaemia, pulmonary edema, and metabolic acidosis [4, 5]. Approximately 6% of AKI patients in the ICU require RRT [6], yet the mortality rate can be as high as 50-70% [4, 7, 8]. Some studies have even suggested that patients undergoing RRT may face an elevated risk of adverse events, including mortality, compared to those not receiving such treatment [9, 10]. CRRT is a blood purification technique primarily used for the treatment of AKI, systemic inflammatory response syndrome, and multiple organ dysfunction syndrome, among other critical illnesses. For AKI patients undergoing CRRT, the mortality rate is greater in the early stages, but it levels off as treatment progresses [11, 12]. This suggests that the short-term and long-term prognoses of these patients may be affected by different risk factors. Therefore, the precise identification of early and late mortality risks in patients undergoing CRRT is profoundly beneficial for clinicians.

Traditional statistical methods such as logistic regression (LR) and the Cox proportional hazards model can be used to predict the development of AKI. Yao et al. [13] employed logistic regression to construct a nomogram model for predicting the risk of in-hospital mortality associated with AKI, demonstrating commendable predictive accuracy. Additionally, Hu, Peng, and their team employed Cox regression to predict the progression of sepsis-induced AKI patients, also exhibiting commendable predictive efficacy [14, 15]. The diagnosis and treatment of kidney diseases pose significant challenges due to the nonlinear, intricate, and variable pathophysiology of the kidney. This complexity makes the application of linear statistical methods problematic. Nonetheless, LR inherently processes the relationship between independent and dependent variables linearly, potentially leading to an oversimplification of intricate nonlinear interactions. Moreover, LR is prone to the influence of multicollinearity among variables, which may impede the model’s efficacy. Hence, the pursuit of more robust and precise predictive instruments is critically vital for the efficacious management of AKI. Currently, machine learning (ML) algorithms have shown promise for the early detection and accurate prediction of AKI progression [16, 17]. ML-powered decision support may help solve these difficulties and improve the clinical and research outcomes of nephrology [18, 19]. To date, no research has focused on developing risk classifiers for CRRT patients at different time points. ML algorithms may automatically identify and anticipate the progression of AKI in multivariate patient data. This can help clinicians identify AKI patients at high risk of mortality and enable early therapeutic actions that may improve patient outcomes. Therefore, our study aimed to use ML algorithms to construct a risk stratification tool and detect AKI patients for whom the initiation of CRRT was associated with poor survival outcomes at 10 and 90 days. This can help clinicians realize individualized differentiation and decision-making for AKI patients undergoing CRRT.

## Method

### Data sources and participants

The data of this retrospective study were derived from two sets. The Dryad database, which contains the medical records of AKI patients who received CRRT in the ICU at Yonsei University Health System Severance Hospital and National Health Insurance Service Medical Center Ilsan Hospital, provided all the data for the training set [20]. Since the original study subjects were deidentified, no written consent was required from the recorded patients. The data for the validation set came from the MIMIC-IV database. The MIMIC database is a free database for clinical researchers worldwide [21]. From 2008 to 2019, more than 38,000 people who were hospitalized at Beth Israel Deaconess Medical Center in Boston, Massachusetts, were tracked. We completed the training course at the National Institutes of Health and obtained the certificate (Completion Record ID: 48,551,683). This study obtained permission for ethical exemption from the ethics committee of the Affiliated Hospital of Nanjing University of Traditional Chinese Medicine. Patients with stage 2–3 AKI (defined as at least a 2-fold increase in serum creatinine) who underwent CRRT were enrolled in this study. Because information was not recorded before admission in the MIMIC-IV database, we used the baseline creatinine level after admission as a reference. Patients who were under the age of 18, had insufficient laboratory tests (missing values greater than 30%), had a history of advanced chronic kidney disease (defined as an eGFR < 15 mL/min/1.72 m^2^) or who underwent RRT on the day or before admission were excluded from this study.

### Primary outcomes and exposure factors

Short-term mortality was defined as death within 10 days of starting CRRT, while long-term mortality was defined as death within 90 days. Survival time was calculated by subtracting the time of death from the time of hospitalization. Based on our clinical experience and literature review, we selected the following variables as our exposure factors: (1) demographics (gender and age); (2) complications (myocardial infarction, heart failure, cerebrovascular diseases, peripheral vascular disease, diabetes mellitus, hypertension, chronic obstructive pulmonary disease (COPD), and dementia); (3) laboratory parameters (potassium, bicarbonate, serum phosphate, white blood cell (WBC), hemoglobin, blood urea nitrogen (BUN), serum albumin, and estimated glomerular filtration rate (eGFR, based on the CKD-EPI formula)); (4) physical parameters (body mass index (BMI), systolic pressure, and diastolic pressure)); and (5) other variables (mechanical ventilation, two-hour urine output, SOFA scores, the renal reactive renal replacement therapy (CRRT) dose, AKI stage (according to the Kidney Disease: Improving Global Outcomes (KDIGO) guidelines [22], stage 2: the serum creatinine level was 2.0-2.9 times greater than the baseline; stage 3: the serum creatinine level was 3.0 times greater than the baseline (or an increase in the Scr greater than 353.6 µmol/L)). All of the data from the laboratory and physical exam were analysed using data from the initiation of CRRT.

### Statistical analysis

For the description of participation, we grouped the enrolled patients into two groups (patients who died or not) according to their survival status. The Shapiro‒Wilk test was applied to assess the normality of the distribution of continuous data. Using Student’s t test, continuous variables that fit a normal distribution were compared, and the results are shown as the mean ± standard deviation (SD). The nonnormally distributed variables were then identified using the Kruskal‒Wallis (KW) test, and they are displayed as the median (1st–3rd quartile). Chi-square tests were performed to compare categorical variables. If the theoretical frequency was less than 10, Fisher’s exact test was used. For variables with less than 30% missing values, we employ the random forest algorithm, and for variables with more than 30% missing values, we choose to delete them.

### Construction of the machine learning model

Nine different types of ML algorithms were utilized in this study to estimate the likelihood of 10- and 90-day mortality: extreme gradient boost (XGBoost), logistic, light gradient boosting machine (LightGBM), random forest, adaptive boost (AdaBoost), Gaussian naive Bayes (GausianNB), multilayer perceptron (MLP), support vector machine (SVM), and k-nearest neighbor (KNN) methods. (Supplement Table 1)The training set was used for constructing the model. As part of the training process, 5-fold cross-validation was utilized to identify the optimum hyperparameters and avoid overfitting. The generalizability of the ML model was examined using the validation set. Several metrics (area under the receiver operating characteristic curve (AUROC), F1 score, accuracy, sensitivity, specificity, and precision) were used to assess the predictive performance of various ML models [23]. An AUROC closer to 1 indicates that the classifier has good discrimination and prediction value. The best-performing classifier was determined by the metrics of the validation set. The statistical analyses were performed using R software version 4.05 (https://www.rproject.org/), and *P* < 0.05 (double) was considered to indicate statistical significance.

**Table 1:** Comparison of risk factors of 10 days mortality after initiation of continuous renal replacement therapy (CRRT)

Variates	Survival (*n* = 1015)	Death (*n* = 520)	*p*-value
Age (yr)	65.0 (53.0–73.0)	65.0 (53.0–73.0)	0.593
Gender(male)	618 (60.9%)	326 (62.7%)	0.527
Miocardial infarction	122 (12.0%)	53 (10.2%)	0.326
Congestive heart failure	221 (21.8%)	70 (13.5%)	< 0.001
Cerevascular diseases	106 (10.4%)	41 ( 7.9%)	0.128
Peripheral vascular disease	37 ( 3.6%)	34 ( 6.5%)	0.015
Dementia	42 ( 4.1%)	26 ( 5.0%)	0.518
Diabetes mellitus	350 (34.5%)	163 (31.3%)	0.24
Hypertension	658 (64.8%)	253 (48.7%)	< 0.001
COPD	66 ( 6.5%)	82 (15.8%)	< 0.001
Mechanical ventilation	744 (73.3%)	457 (87.9%)	< 0.001
Potassium (mmol/L)	4.5 (4.0-5.1)	4.5 (3.9–5.4)	0.131
Bicarbonate (mmol/L)	17.0 (14.0–20.0)	17.0 (13.0–21.0)	0.479
Phosphorous (mmol/L)	5.2 (3.9–6.5)	5.8 (4.7–7.5)	< 0.001
Body mass index (Kg/m^2^)	23.7 (21.1–26.3)	23.7 (20.8–26.2)	0.5
Systolic pressure (mmHg)	113.0 (100.0-129.0)	106.0 (93.0-117.0)	< 0.001
Diastolic pressure (mmHg)	61.0 (51.0–70.0)	57.0 (49.0–66.0)	< 0.001
WBC	12630.0 (7960.0-18850.0)	11095.0 (4660.0-17287.5)	< 0.001
Hemoglobin (g/L)	9.4 (8.4–10.8)	9.2 (8.1–10.3)	0.001
Blood urea nitrogen (mmol/L)	48.0 (33.0–70.0)	52.0 (33.0-76.2)	0.172
Albumin (g/L)	2.7 (2.3-3.0)	2.5 (2.2–2.9)	< 0.001
eGFR (ml/min per 1.73 m^2^)	24.5 (15.7–38.1)	29.0 (19.8–38.2)	< 0.001
Urine output (ml/2 h)	40.0 (10.0-110.0)	20.0 (0.0–75.0)	< 0.001
SOFA scores	12.0 (9.0–14.0)	14.0 (12.0–16.0)	< 0.001
CRRT_dose (ml)	36.9 (34.5–39.9)	36.9 (33.3–39.9)	0.091
AKI stage	249 (24.5%)	119 (22.9%)	0.514
	766 (75.5%)	401 (77.1%)	

Abbreviation: COPD: chronic obstructive pulmonary disease; WBC: white blood cell; eGFR: estimated glomerular filtration rate; AKI: acute kidney injury. The category risk factors are shown as n (%), while continuous risk factors are shown as median (1th -3th )

## Results

### Baseline characteristics

According to our inclusion criteria, a total of 1535 patients were included in this study. (Table 1). Among them, 1144 patients were in the training set, and 391 patients were in the validation set (Supplement Table 2). The median age of the patients in the training cohort was 66 (54–74) years; 61.6% were men; 26% (419) had stage 2 AKI; the median eGFR was 26.6 (17.2–38.5) ml/min/1.73 m2, and the median SOFA score was 12 (10–14). The median age of the patients in the validation group was 63 (50–70) years, 61.1% were men, 17.9% had stage 2 AKI, the median eGFR was 25.5 (16.6–36.8) ml/min/1.73 m2, and the median SOFA score was 13 (10–15).

**Table 2:** Comparison of risk factors of 90 days mortality after initiation of continuous renal replacement therapy (CRRT)

Variates	Survival (*n* = 455)	Death (*n* = 1080)	*p*-value
Age (yr)	65.0 (51.5–72.0)	65.0 (53.8–73.0)	0.29
Gender(male)	270 (59.3%)	674 (62.4%)	0.285
Miocardial infarction	54 (11.9%)	121 (11.2%)	0.775
Congestive heart failure	89 (19.6%)	202 (18.7%)	0.749
Cerevascular diseases	49 (10.8%)	98 ( 9.1%)	0.349
Peripheral vascular disease	17 ( 3.7%)	54 ( 5.0%)	0.345
Dementia	20 ( 4.4%)	48 ( 4.4%)	0.99
Diabetes mellitus	170 (37.4%)	343 (31.8%)	0.039
Hypertension	313 (68.8%)	598 (55.4%)	< 0.001
COPD	36 ( 7.9%)	112 (10.4%)	0.163
Mechanical ventilation	299 (65.7%)	902 (83.5%)	< 0.001
Potassium (mmol/L)	4.5 (4.0–5.0)	4.5 (4.0-5.2)	0.45
Bicarbonate (mmol/L)	17.0 (14.0–20.0)	17.0 (14.0–20.0)	0.876
Phosphorous (mmol/L)	5.2 (3.9–6.2)	5.6 (4.3-7.0)	< 0.001
Body Mass index (Kg/m^2^)	24.1 (21.5–26.8)	23.5 (20.8–25.9)	0.002
Systolic pressure (mmHg)	115.0 (102.0-132.0)	108.0 (95.0-121.0)	< 0.001
Diastolic pressure (mmHg)	61.0 (52.0–71.0)	58.0 (49.0–68.0)	< 0.001
WBC	13720.0 (9010.0-19420.0)	11355.0 (5400.0-17860.0)	< 0.001
Hemoglobin (g/L)	9.6 (8.5–11.0)	9.3 (8.2–10.4)	< 0.001
Blood urea nitrogen (mmol/L)	47.0 (32.5–63.0)	52.0 (34.0-75.2)	0.001
Albumin (g/L)	2.8 (2.4–3.2)	2.5 (2.2–2.9)	< 0.001
eGFR (ml/min per 1.73 m^2^)	22.0 (15.1–35.5)	28.0 (17.9–39.0)	< 0.001
Urine output (ml/2 h)	55.0 (13.5–120.0)	30.0 (2.0–85.0)	< 0.001
SOFA scores	10.0 (8.0–13.0)	13.0 (11.0–15.0)	< 0.001
CRRT_dose (ml)	36.8 (34.3–39.8)	37.0 (34.1–40.0)	0.592
AKI stage	103 (22.6%)	265 (24.5%)	0.465
	352 (77.4%)	815 (75.5%)	

Abbreviation: COPD: chronic obstructive pulmonary disease; WBC: white blood cell; eGFR: estimated glomerular filtration rate; AKI: acute kidney injury. The category risk factors are shown as n (%), while continuous risk factors are shown as median (1th -3th )

### Comparison of risk factors for 10-day mortality after initiation of CRRT

A total of 520 (33.9%) patients died after the initiation of CRRT within 10 days. Table 1 shows that patients who died within 10 days had lower rates of congestive heart failure (13.5%/21.8%) and hypertension (48.7%/64.8%) (*P* < 0.01) and greater rates of peripheral vascular disease (6.5%/3.6%), COPD (15.8%/6.5%), and mechanical ventilation (87.9%/73.3%) (*P* < 0.01). Those who died within 10 days had greater phosphorus (5.8/5.2 mmol/L), eGFR (29/24.5 ml/min/1.73 m^2^), and SOFA scores (14/12) and lower systolic pressure (106/113 mmHg), diastolic pressure (57/61 mmHg), haemoglobin (9.2/9.4 g/L), albumin (2.5/2.7 g/L), and urine output (20/40 ml/2 h) (*P* < 0.01).

### Comparison of risk factors for 90-day mortality after initiation of CRRT

A total of 1080 (68.8%) patients died within 90 days after the initiation of CRRT. Table 2 shows that patients who died within 90 days had a lower rate of diabetes (31.8%/37.4%) and hypertension (55.4%/68.8%) (*P* < 0.05) and a higher rate of mechanical ventilation (83.5%/65.7%) (*P* < 0.01). Those who died within 90 days had higher levels of phosphorus (5.6/5.2 mmol/L), eGFR (28/22 ml/min per 1.73 m^2^), and BUN (52/47 mmol/L) and SOFA scores (13/10) and lower BMIs (23.5/24.1 kg/m^2^), systolic pressure (108/115 mmHg), diastolic pressure (58/61 mmHg), leukocyte count (11,355/13,720), haemoglobin (9.3/9.6 g/L), albumin (2.5/2.8 g/L), and urine output (30/55 ml/2 h) (*P* < 0.01).

### The prediction performance of the established model on the validation set (10 days)

**Fig. 1:**
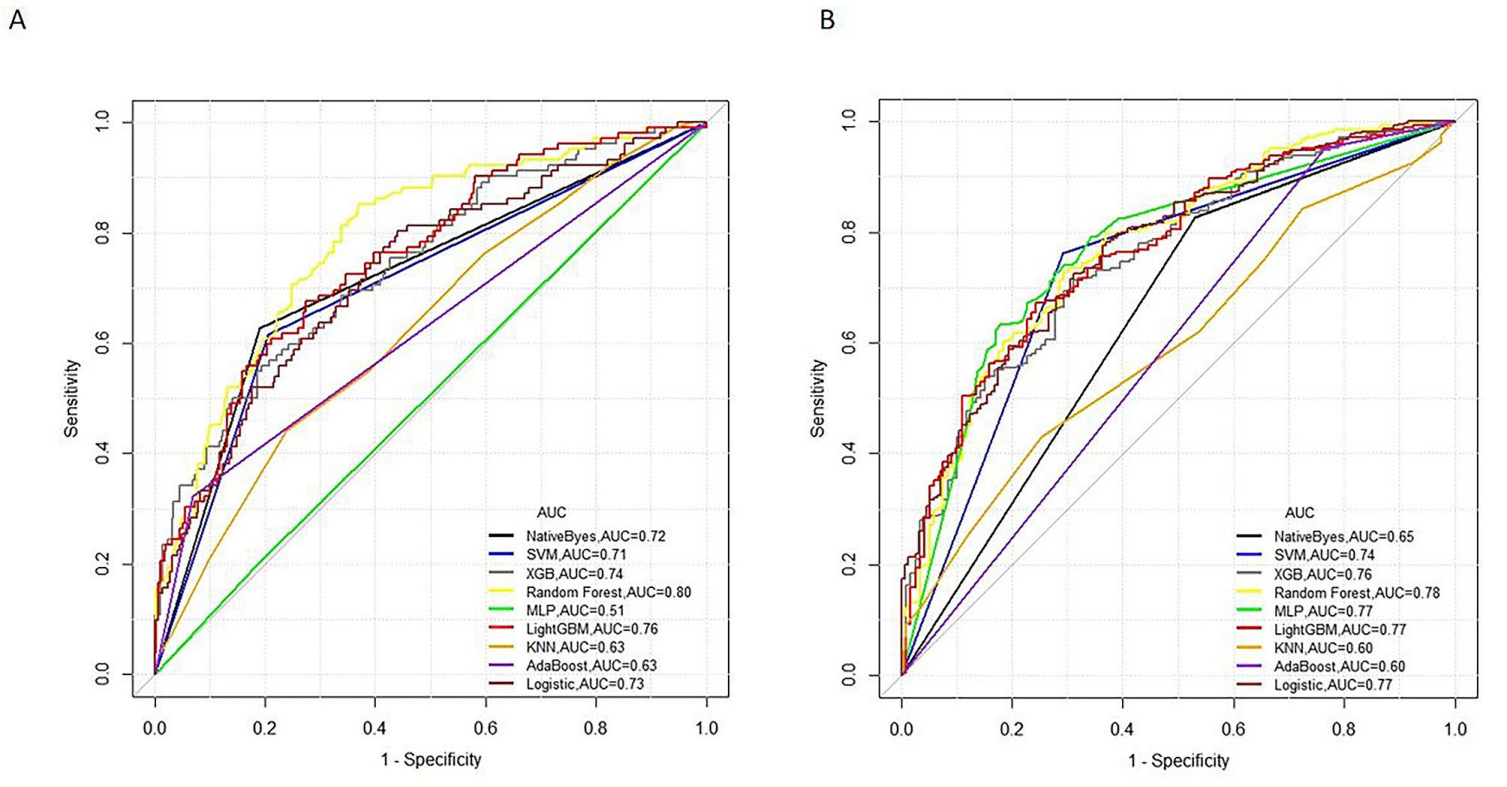
The receiver operating characteristic (ROC) curves of each model. **(A)** 10 days; **(B)** 90 days. Abbreviations: SVM: support vector machine; XGB: extreme gradient boost; MLP: multilayer perceptron; KNN: k-nearest neighbor

**Fig. 2:**
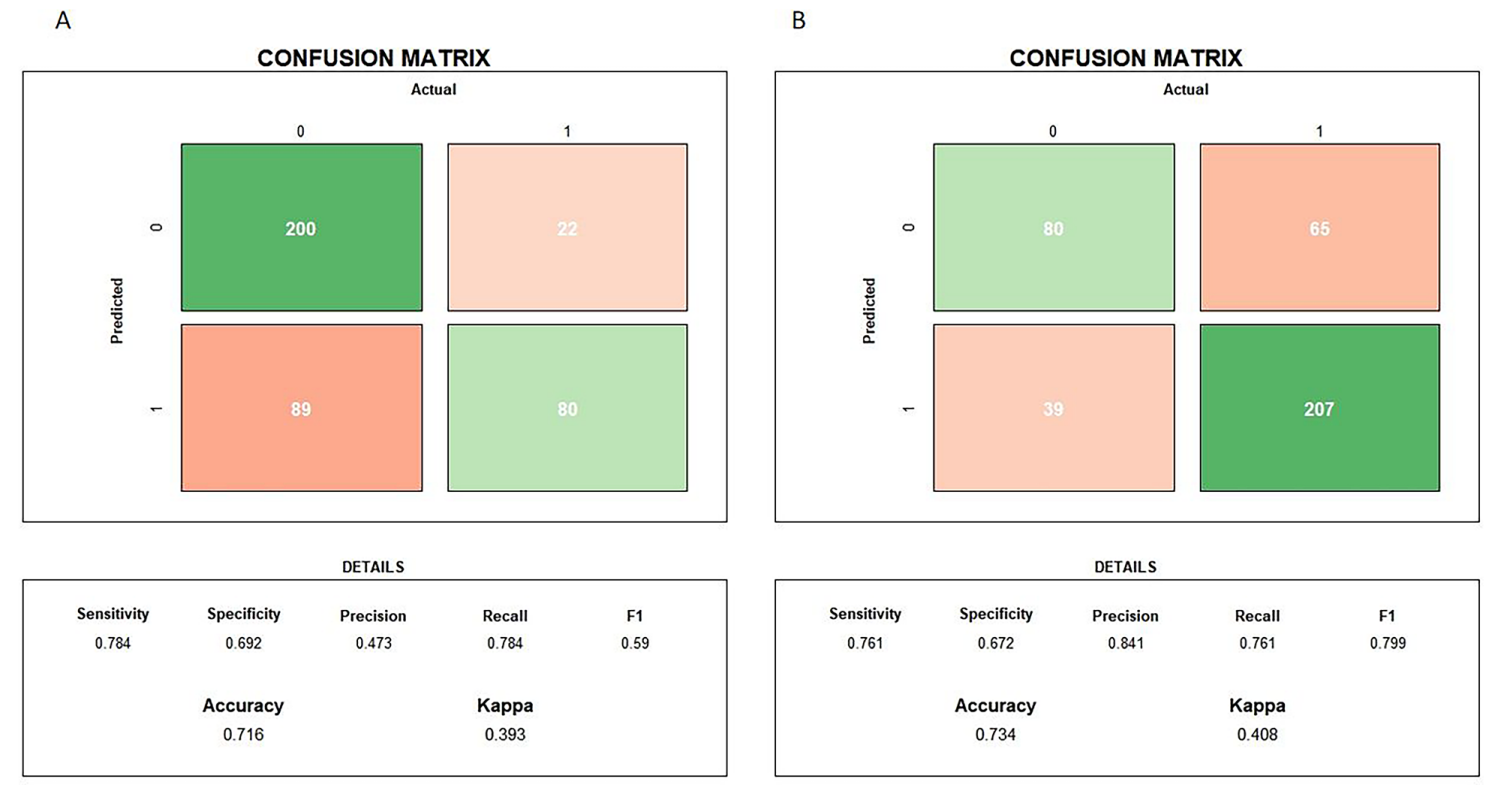
The confusion matrix of the validation set. **A**. 10 days; **B**. 90 days 1: positive outcome (death); 0: negative outcome (survival)

**Fig. 3:**
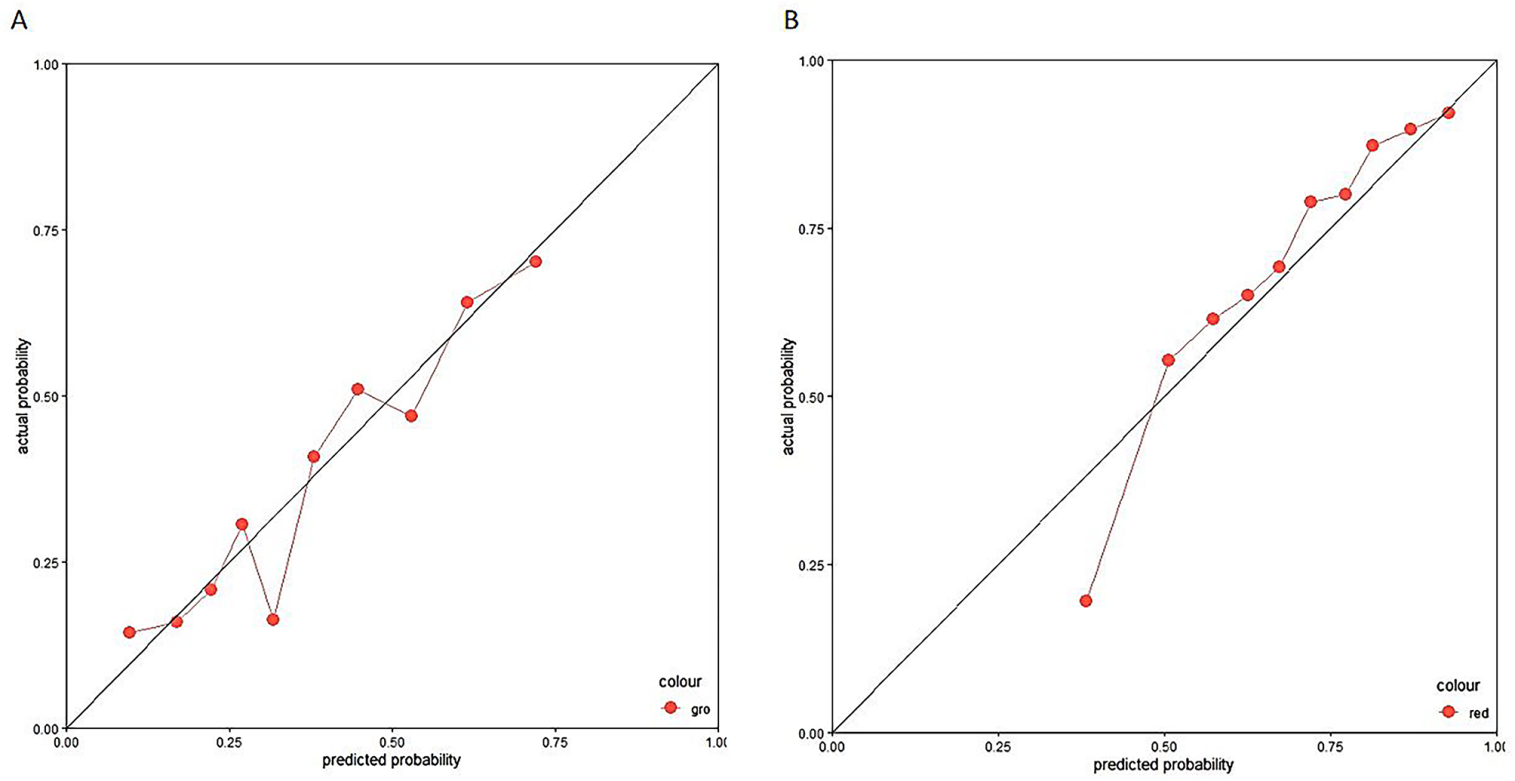
The calibration curve of the random forest model. **A**. 10 days; **B**. 90 days

Patients who died within 10 days after the initiation of CRRT were labeled as class 1, whereas those who survived after 10 days were grouped as 0. Then, we developed multiple classifiers with different ML algorithms based on training sets. The validation set was used to test the generalizability of the constructed model OR was used to test the predictive power of the model. The AUROCs of the various models ranged from 0.51 to 0.80. Among the various ML algorithms, the RF model had the best prediction performance (Supplement Table 3) on the validation set (AUROC: 0.80, 95% CI: 0.74–0.84; Accuracy: 0.72, 95% CI: 0.67–0.76; F1-score: 0.59), while the MLP model had the weakest predictive performance (AUROC: 0.51, 95% CI: 0.31–0.70; Accuracy: 0.634, 95% CI: 0.584–0.682; F1-score: 0.41). Figure 1 shows the ROC curves of each model for mortality prediction. The classification results of the RF model for the validation set can be seen in the confusion matrix (Fig. 2), with 200 of the predictions being correctly negative and 80 being correctly positive. The calibration curve (Fig. 3) demonstrated that the difference between the predicted and observed risks fluctuated around the diagonal, indicating that the predicted risk of short-term mortality was generally consistent with the observed risk.

### The prediction performance of the established model on the validation set (90 days)

The AUROC of the various ML models ranged from 0.60 to 0.78. The RF model had the best prediction performance (Supplement Table 4) on the validation set (AUROC: 0.78, 95% CI: 0.73–0.83; Accuracy: 0.73, 95% CI: 0.69–0.78; F1-score: 0.80), while the AdaBoost and KNN models had the worst (AUROC: 0.60, 95% CI: 0.55–0.63; Accuracy: 0.73, 95% CI: 0.69-0.72-0.78; F1-score: 0.83). Figure 1 shows the ROC curves of each model for mortality prediction. The confusion matrix (Fig. 2) shows the classification results of the RF model for the validation set, of which 80 correctly predicted negative and 207 correctly predicted positive results. The calibration curve (Fig. 3) revealed that the predicted risk of short-term mortality was generally consistent with the observed risk. We utilized grid search for hyperparameter tuning, and the hyperparameter values used for all machine learning algorithms are provided in Supplement Table 5.

### Important features

**Fig. 4:**
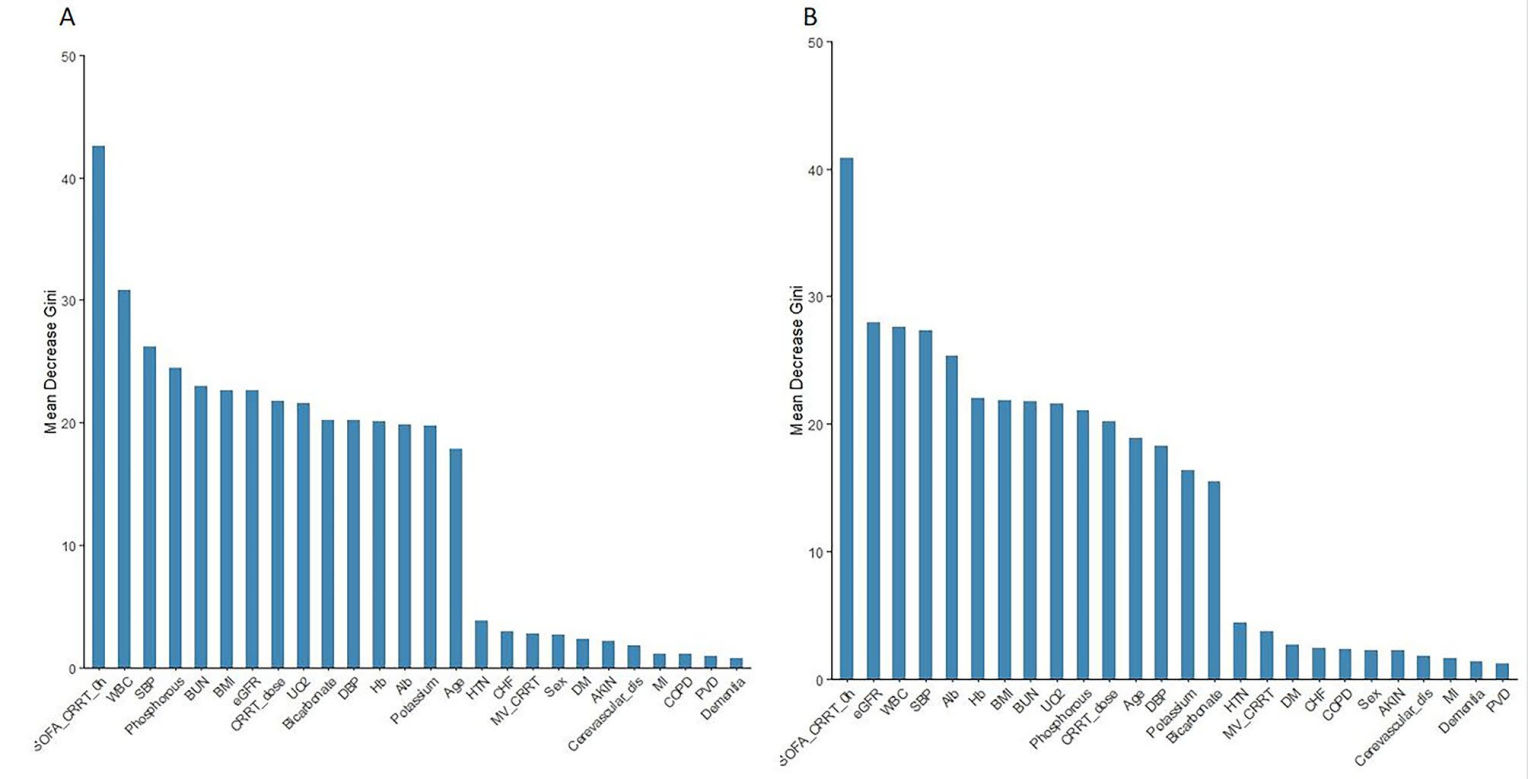
Ranking of feature importance according to the mean decreased GINI impurity. **A**. 10 days; **B**. 90 days

We utilized the mean decrease in Gini impurity to assess the extent to which risk factors contributed to short-term and long-term mortality. According to Fig. 4, the SOFA score was the main contributing factor to short-term mortality in CRRT patients. The five risk factors for short-term death were also WBC, systolic blood pressure, phosphorus, and BUN. For long-term mortality, SOFA scores remained the most important risk factor contributing to long-term mortality in CRRT patients. However, the order of the top five risk factors changed to eGFR, WBC, systolic blood pressure, and serum ALB.

### SHAP analysis

**Fig. 5:**
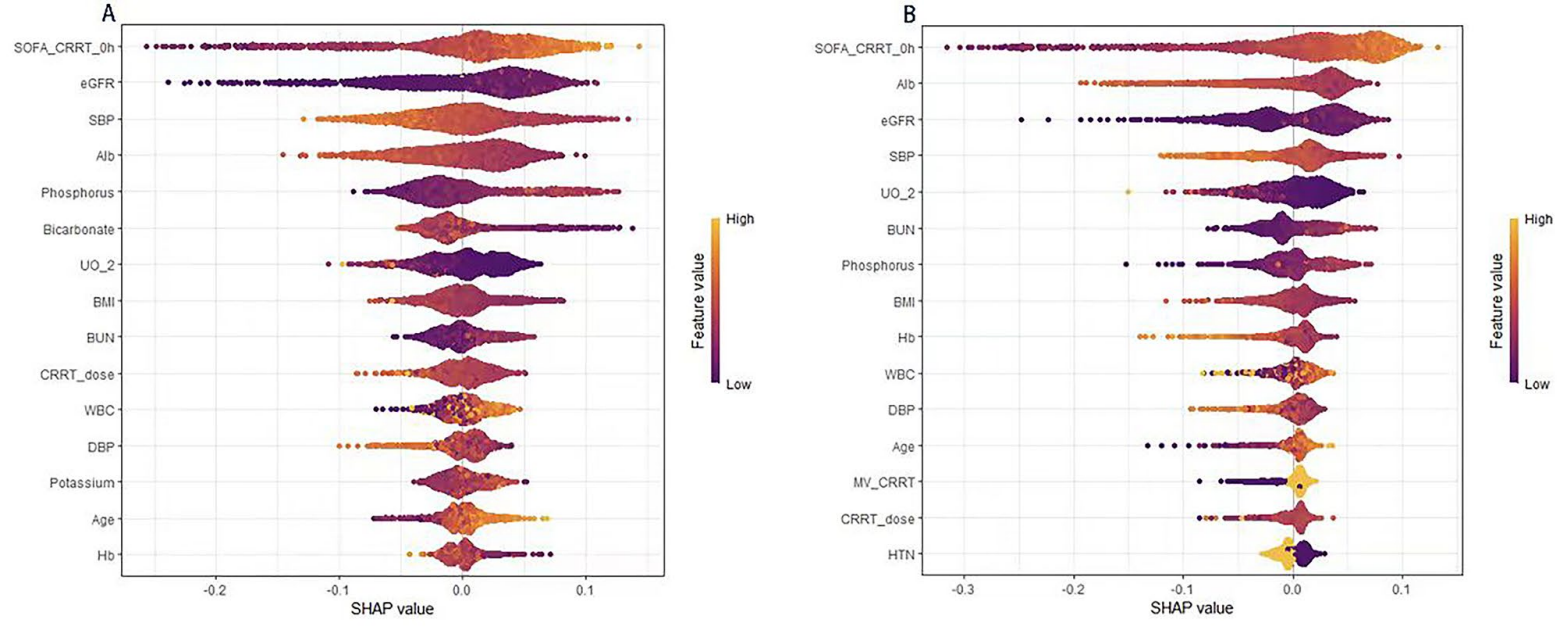
SHAP Value Plot. **A**. 10 days; **B**. 90 days. Feature values are represented from low (purple) to high (yellow), with each feature’s impact on mortality risk increasing from left to right

SHAP (SHapley Additive exPlanation) is a technique used to interpret the outputs of machine learning models. Figure 5 illustrates the SHAP values for the Random Forest model, which demonstrated the best performance in predicting outcomes. Each point represents a patient. The x-axis shows the SHAP values, indicating the impact of each feature on the prediction. Points to the right indicate an increased probability of death, while points to the left indicate a decreased probability. The y-axis lists all the features, and the color gradient from purple to yellow indicates the feature values from low to high, with dark purple representing low values and yellow representing high values. For both short-term (10-day) and long-term (90-day) mortality risk predictions, the SOFA score is the most critical predictor. High SOFA scores (shown in yellow) significantly increase the likelihood of death, reflecting the severity of organ failure on patient prognosis. Low eGFR values (shown in purple) are strongly associated with a higher risk of death, underscoring the importance of renal function in patient survival. Low serum ALB is linked to higher mortality risk across both time frames, indicating that malnutrition adversely affects both short-term and long-term survival. Systolic blood pressure (SBP) shows a significant correlation between low blood pressure and increased mortality risk, consistent across both periods, highlighting the importance of blood pressure management in improving patient survival. Elevated levels of phosphorus and BUN contribute significantly to the increased long-term (90-day) mortality risk, indicating metabolic imbalances and organ dysfunction in patients.

## Discussion

Patients with AKI who undergo CRRT are at high risk of mortality. In our study, at 10 days and 90 days following the initiation of CRRT, the mortality rates were 33.1% and 66.8%, respectively. This finding is in line with the results of a secondary analysis of two multicenter randomized controlled trials (AKIKI and IDEALICU) [24]. Given that the mortality rates for the short and long term are different and that there may be different risk factors that have contributed to the mortality rates for these two time periods, risk stratification tools for two distinct periods of mortality should be established. To our knowledge, our study is the first to focus on constructing risk stratification tools and investigating risk factors for different periods of mortality in AKI patients after the initiation of CRRT. The results of our study show that the RF model is the most accurate algorithm for predicting both short-term and long-term mortality rates. (10 days: ROC: 0.80, 95% CI: 0.74–0.84; 90 days: ROC: 0.78, 95% CI: 0.73–0.83). Previously, Daniel H. Li [25] constructed a prediction model based on traditional logistic regression to predict the risk of long-term (90-day) mortality in AKI patients who were undergoing RRT. Nonetheless, that study had poor predictive performance (AUC: 0.61, 95% CI: 0.54–0.69) on external validation, which indicates that the external applicability of the model is poor. Zheng-hai Bai et al. [26] developed a nomogram for predicting mortality risk 28 days after the initiation of CRRT using multifactor Cox regression analysis, which has similar predictive performance (area under the curve (AUC): 0.78, 95% CI: 0.75–0.82) to that of our RF model. However, this study did not conduct external validation to verify the generalizability of the established model, so its external application at other centers is unclear. Compared with these studies, our research examined the predictive ability of various ML models and revealed that the RF model outperforms traditional statistical analysis in forecasting both short-term and long-term mortality, and this performance was maintained through external validation.

Our study focused on predicting mortality risk in the CRRT modality rather than in the intermittent haemodialysis (IHD) modality since several studies based on real-world data indicated that patients undergoing CRRT were at a greater risk of death than those receiving intermittent haemodialysis (IHD) [24, 27]. An accurate risk stratification tool for these patients is needed. Interestingly, we selected 10-day and 90-day mortality periods. Earlier research [12] showed that the death rate was highest in the first 10 days after the initiation of CRRT, and from 10 days to 90 days, the risk of death gradually decreased and remained at a low level thereafter. Therefore, these two time periods may be important nodes for patient stratification. By identifying patients who are at a high risk of short-term death, clinicians can execute essential therapies to help them pass this period, and then, the prognosis of these patients will be considerably improved.

The ranking of important features was performed using the RF model after we determined that it was the best prediction algorithm. Notably, the SOFA score was the top risk factor for both 10-day mortality and 90-day mortality according to the feature importance ranking, indicating that it is an important prognostic factor for both short-term and long-term mortality risk evaluation. This result is also consistent with previous traditional statistical analyses [28–30]. Several studies have investigated the predictive value of SOFA scores for early and long-term death risk in AKI patients undergoing CRRT. Using stepwise logistic regression, Herreweghe et al. reported that higher SOFA scores were associated with 2-day mortality after RRT initiation [12]. Daniel H. Li [25] used a stepwise model and reported that SOFA scores were associated with 90-day mortality after RRT initiation. Combined with our study, SOFA scores should be recommended as a key predictive factor for predicting mortality risk (regardless of early or long-term mortality) in AKI patients initiated with CRRT. In addition to SOFA score, the WBC count is also an important risk factor for both short-term and long-term mortality. It has previously been shown that WBCs are the primary cause of in-hospital mortality for patients with AKI who are receiving CRRT [31]. However, this study did not take into account the risk of short-term mortality. Our research, on the other hand, demonstrated that WBC counts are an excellent predictor of short-term mortality. This may be due to the greater likelihood of early death in AKI patients due to infection. It is also worth mentioning that the eGFR may be more useful for predicting long-term mortality than for predicting short-term mortality. We hypothesize that this may be because a higher eGFR is associated with long-term AKI recovery and a lower rate of dialysis dependency. A single-center retrospective study also revealed that a lower eGFR was associated with long-term renal and overall survival [32]. Aside from those listed above, other risk factors, such as the serum ALB or phosphorus concentration, may also have different effects on short-term and long-term mortality. This also shows the importance of developing classification tools for both short-term and long-term mortality.

The limitations of our study should be acknowledged. For starters, the patients who were included were already undergoing CRRT, and those who were not were not included in the comparisons. Consequently, we can only determine the mortality risk of these individuals, but we have no idea if CRRT will be beneficial to them. Second, our data mostly originate from an electronic medical records database, which is a retrospective study, and the level of evidence is lower than that of a prospective study. Therefore, the model’s external applicability still has to be tested in future investigations. Our research objective is to predict the mortality risk of AKI patients undergoing CRRT at different time points (10 days and 90 days). To achieve this, we utilized two separate classification analysis models. However, we also recognize that survival analysis might be a more intuitive choice as it can generate a risk curve over time (such as the Kaplan Meier curve) for each patient. Although survival analysis might provide more comprehensive information, we opted for classification analysis primarily due to its simplicity and intuitiveness, which are especially important for clinicians. In our 90-day risk model, approximately half of the “death” cases actually died within 10 days. This might suggest that this model is not identifying risk factors for long-term mortality. Nevertheless, we chose to include all death cases because our goal is to identify the mortality risk of all AKI patients, regardless of whether they die within the first 10 days of CRRT. However, we also acknowledge that if a patient survives the first 10 days of CRRT, excluding early deaths could make the 90-day prediction model more accurately reflect the risk factors for long-term mortality. We also noted that three of the top five prognostic factors for short-term and long-term mortality are shared, which might reflect the central role of these factors in the mortality risk of AKI patients. This might suggest that while some risk factors are consistently important throughout the disease process, other risk factors might only play a role at specific time intervals. These differences could have significant implications for clinical decision-making and patient management.

## Conclusion

In this study, we used various ML algorithms to develop two risk stratification tools for identifying AKI patients with a high mortality risk following the commencement of CRRT. The findings suggest that the risk factors for various periods of mortality may be distinct. In terms of both short-term and long-term mortality, the main causes of death were SOFA score and WBC count. The RF algorithm has the best performance in regard to prediction, and this performance remains high even when it is subjected to external validation. In the future, additional external validation needs to be carried out to verify the external application of this model.

### Electronic supplementary material

Below is the link to the electronic supplementary material.

Supplementary Material 1

Supplementary Material 2

Supplementary Material 3

Supplementary Material 4

Supplementary Material 5

## Data Availability

The datasets used and analysed in this study are available from the first author and corresponding author on reasonable request.
