# Supplementary material for: Using machine learning to predict the risk of short-term and long-term death in acute kidney injury patients after commencing CRRT
Source: BMC Nephrol. 2024 Jul 30;25:245. doi: 10.1186/s12882-024-03676-x (PMC11289973; doi:10.1186/s12882-024-03676-x)
Supplement: Supplementary file 1 — Supplementary Material 1 [file 12882_2024_3676_MOESM1_ESM.docx]

| Method | Introduction | Advantages | Disadvantages |
| --- | --- | --- | --- |
| XGBoost | XGBoost is an optimized gradient boosting machine algorithm, designed to improve speed and performance. | Efficient, flexible, customizable, has good performance. | Sensitive to parameter selection, may require a lot of tuning and validation. |
| Logistic Regression | Logistic regression is a statistical model used for binary classification problems. | Simple, fast, easy to understand, output can be interpreted as probability. | Assumes a linear relationship between features and output, may not handle complex relationships. |
| LightGBM | LightGBM is a gradient boosting machine algorithm based on tree, designed to improve efficiency and speed. | Efficient, can handle large-scale data, has good performance. | Sensitive to parameter selection, may require a lot of tuning and validation. |
| Random Forest | Random forest is an ensemble learning method based on decision trees. | Powerful, flexible, can handle various types of data, has good performance. | May overfit, sensitive to outliers. |
| AdaBoost | AdaBoost is an adaptive boosting algorithm, improving performance by combining multiple weak learners. | Powerful, flexible, can be used with various types of learners. | Sensitive to noise and outliers, may overfit. |
| Gaussian naive bayes | GaussianNB is a classification algorithm based on Bayes’ theorem, assuming features follow Gaussian distribution. | Simple, fast, suitable for high-dimensional data. | Assumes features are independent of each other, which may not hold in practice. |
| Multi-layer perceptron | MLP is a feedforward neural network, can be used for classification and regression problems. | Powerful, flexible, can handle complex nonlinear relationships. | Training may take a long time, sensitive to parameter selection. |
| Support vector machine | SVM is a classification and regression algorithm, separating data by finding a hyperplane that maximizes the margin. | Powerful, flexible, has good performance. | Low efficiency in handling large-scale data, sensitive to parameter selection. |
| K-nearest neighbor | KNN is an instance-based learning algorithm, predicting new instance labels by looking at the k nearest neighbors. | Simple, intuitive, easy to understand. | High computational cost, low efficiency in handling large-scale data. |

**Supplement table 1. Introduction to different types of machine learning algorithms.**
