# Supplementary material for: Using machine learning to predict the risk of short-term and long-term death in acute kidney injury patients after commencing CRRT
Source: BMC Nephrol. 2024 Jul 30;25:245. doi: 10.1186/s12882-024-03676-x (PMC11289973; doi:10.1186/s12882-024-03676-x)
Supplement: Supplementary file 2 — Supplementary Material 2 [file 12882_2024_3676_MOESM2_ESM.docx]

| Variates | Training set (n=1144) | Validation set (n=391) | p-value |
| --- | --- | --- | --- |
| Age (yr) | 66.0 (54.0-74.0) | 63.0 (50.0-70.0) | <0.001 |
| Gender（meal） | 705 (61.6%) | 239 (61.1%) | 0.908 |
| Miocardial infarction | 112 ( 9.8%) | 63 (16.1%) | 0.001 |
| Congestive heart failure | 186 (16.3%) | 105 (26.9%) | <0.001 |
| Cerevascular diseases | 118 (10.3%) | 29 ( 7.4%) | 0.114 |
| Peripheral vascular disease | 46 ( 4.0%) | 25 ( 6.4%) | 0.074 |
| Dementia | 42 ( 3.7%) | 26 ( 6.6%) | 0.02 |
| Diabetes mellitus | 399 (34.9%) | 114 (29.2%) | 0.045 |
| Hypertension | 601 (52.5%) | 310 (79.3%) | <0.001 |
| COPD | 85 ( 7.4%) | 63 (16.1%) | <0.001 |
| Mechanical ventilation | 897 (78.4%) | 304 (77.7%) | 0.84 |
| Potassium (mmol/L) | 4.5 (4.0-5.2) | 4.4 (3.9-5.0) | 0.01 |
| Bicarbonate (mmol/L) | 17.0 (13.0-20.0) | 17.9 (15.8-20.0) | 0.001 |
| Phosphorous (mmol/L) | 5.4 (4.1-6.9) | 5.4 (4.2-6.7) | 0.981 |
| Body Mass index (Kg/m^2^) | 23.7 (20.9-26.2) | 23.7 (20.9-26.6) | 0.591 |
| Systolic pressure (mmHg) | 110.0 (97.8-125.0) | 110.0 (99.0-124.0) | 0.58 |
| Diastolic pressure (mmHg) | 60.0 (50.0-69.2) | 58.0 (49.0-66.0) | 0.005 |
| Leukocyte count | 11830.0 (6517.5-18532.5) | 12990.0 (7540.0-18305.0) | 0.272 |
| Hemoglobin (g/L) | 9.4 (8.3-10.7) | 9.3 (8.3-10.4) | 0.448 |
| Blood urea nitrogen (mmol/L) | 49.4 (33.0-73.0) | 48.0 (33.0-69.0) | 0.677 |
| Albumin (g/L) | 2.6 (2.2-3.0) | 2.7 (2.3-3.1) | 0.015 |
| eGFR (ml/min per 1.73 m^2^) | 26.6 (17.2-38.5) | 25.5 (16.6-36.8) | 0.257 |
| Urine output (ml/2h) | 35.0 (5.0-100.0) | 34.0 (4.0-99.0) | 0.747 |
| SOFA scores | 12.0 (10.0-14.0) | 13.0 (10.0-15.0) | 0.001 |
| CRRT_dose (ml) | 36.7 (34.1-39.5) | 38.2 (34.2-40.7) | 0.001 |
| AKI stage | 298 (26.0%) | 70 (17.9%) | 0.001 |
|  | 846 (74.0%) | 321 (82.1%) |  |

**Supplement Table 1 Comparison of risk factors of training set and validation set**

Abbreviation: COPD: chronic obstructive pulmonary disease; eGFR: estimated glomerular filtration rate; AKI: acute kidney injury. The category risk factors are shown as n (%), while continuous risk factors are shown as median (1^th^-3^th^).
