# Supplementary material for: Using machine learning to predict the risk of short-term and long-term death in acute kidney injury patients after commencing CRRT
Source: BMC Nephrol. 2024 Jul 30;25:245. doi: 10.1186/s12882-024-03676-x (PMC11289973; doi:10.1186/s12882-024-03676-x)
Supplement: Supplementary file 4 — Supplementary Material 4 [file 12882_2024_3676_MOESM4_ESM.docx]

|  |  |  |  |  |  |  |
| --- | --- | --- | --- | --- | --- | --- |
| Model | AUROC | F1 | Accuracy | Sensitivity | Specificity | Precision |
| XGBoost | 0.76 | 0.81 | 0.73 | 0.86 | 0.44 | 0.78 |
| AdaBoost | 0.6 | 0.83 | 0.73 | 0.95 | 0.23 | 0.74 |
| LightGBM | 0.77 | 0.78 | 0.71 | 0.77 | 0.56 | 0.8 |
| RandomForest | 0.78 | 0.8 | 0.73 | 0.76 | 0.67 | 0.84 |
| Multi-layer perceptron | 0.77 | 0.74 | 0.68 | 0.62 | 0.83 | 0.92 |
| Support vector machine | 0.74 | 0.84 | 0.75 | 0.94 | 0.33 | 0.76 |
| K-nearest neighbor | 0.6 | 0.74 | 0.63 | 0.75 | 0.34 | 0.72 |
| Logistic | 0.77 | 0.78 | 0.71 | 0.72 | 0.69 | 0.85 |
| Gaussian naive bayes | 0.65 | 0.80 | 0.72 | 0.83 | 0.47 | 0.78 |
|  |  |  |  |  |  |  |

**Supplement table 4. The predict performance (90 day) of each model**
