# Supplementary material for: Using machine learning to predict the risk of short-term and long-term death in acute kidney injury patients after commencing CRRT
Source: BMC Nephrol. 2024 Jul 30;25:245. doi: 10.1186/s12882-024-03676-x (PMC11289973; doi:10.1186/s12882-024-03676-x)
Supplement: Supplementary file 5 — Supplementary Material 5 [file 12882_2024_3676_MOESM5_ESM.docx]

| Method | Hyperparameter | Description | Parameter value |
| --- | --- | --- | --- |
| XGBoost | n_estimators | Number of trees | 100 |
|  | learning_rate | Learning rate | 0.1 |
|  | max_depth | Maximum depth of the trees | 6 |
|  | subsample | Fraction of samples used per tree | 0.8 |
|  | colsample_bytree | Fraction of features used per tree | 0.8 |
|  | gamma | Minimum loss reduction for split | 0 |
|  | reg_lambda | L2 regularization term | 1 |
|  | reg_alpha | L1 regularization term | 0 |
| AdaBoost | n_estimators | Number of weak learners | 50 |
|  | learning_rate | Learning rate | 1 |
|  | base_estimator | Type of base learner | DecisionTreeClassifier |
| LightGBM | n_estimators | Number of trees | 100 |
|  | learning_rate | Learning rate | 0.1 |
|  | num_leaves | Maximum number of leaves per tree | 31 |
|  | max_depth | Maximum depth of the trees | -1 (no limit) |
|  | feature_fraction | Fraction of features used per tree | 0.8 |
|  | bagging_fraction | Fraction of samples used per tree | 0.8 |
|  | min_data_in_leaf | Minimum samples per leaf node | 20 |
|  | lambda_l1 | L1 regularization term | 0 |
|  | lambda_l2 | L2 regularization term | 0 |
| Random Forest | n_estimators | Number of trees | 100 |
|  | max_depth | Maximum depth of the trees | None (no limit) |
|  | min_samples_split | Minimum samples for splitting an internal node | 2 |
|  | min_samples_leaf | Minimum samples per leaf node | 1 |
|  | max_features | Maximum number of features used for splits | sqrt (square root of all features) |
|  | bootstrap | Whether to use sampling with replacement | True |
| Multi-layer perceptron | hidden_layer_sizes | Number of neurons in hidden layers | (100,) |
|  | activation | Activation function | relu |
|  | solver | Optimization algorithm | adam |
|  | alpha | L2 regularization term | 0.0001 |
|  | learning rate | Learning rate adjustment method | constant |
|  | max_iter | Maximum number of iterations | 200 |
| Support vector machine | C | Regularization parameter | 1 |
|  | kernel | Type of kernel function | rbf |
|  | gamma | Kernel coefficient | scale |
|  | degree | Degree of polynomial kernel | 3 |
| K-nearest neighbors | n_neighbors | Number of neighbors | 5 |
|  | weights | Weight function for neighbors | uniform |
|  | algorithm | Algorithm for nearest neighbor search | auto |
|  | leaf_size | Size of leaf node | 30 |
|  | p | Power parameter for distance metric | 2 |
| Logistic | penalty | Type of regularization | 12 |
|  | C | Inverse of regularization strength | 1 |
|  | solver | Optimization algorithm | lbfgs |
|  | max_iter | Maximum number of iterations | 100 |
| Gaussian naive bayes | var_smoothing | Smoothing parameter added to the variance | 1e-9 |

**Supplement table 5. A Key Hyperparameters of Machine Learning Algorithms（10 day）**

| Method | Hyperparameter | Description | Parameter value |
| --- | --- | --- | --- |
| XGBoost | n_estimators | Number of trees | 150 |
|  | learning_rate | Learning rate | 0.05 |
|  | max_depth | Maximum depth of the trees | 7 |
|  | subsample | Fraction of samples used per tree | 0.9 |
|  | colsample_bytree | Fraction of features used per tree | 0.7 |
|  | gamma | Minimum loss reduction for split | 0 |
|  | reg_lambda | L2 regularization term | 1 |
|  | reg_alpha | L1 regularization term | 0 |
| AdaBoost | n_estimators | Number of weak learners | 75 |
|  | learning_rate | Learning rate | 0.8 |
|  | base_estimator | Type of base learner | Decision Tree Classifier |
| LightGBM | n_estimators | Number of trees | 200 |
|  | learning_rate | Learning rate | 0.05 |
|  | num_leaves | Maximum number of leaves per tree | 31 |
|  | max_depth | Maximum depth of the trees | -1 (no limit) |
|  | feature_fraction | Fraction of features used per tree | 0.85 |
|  | bagging_fraction | Fraction of samples used per tree | 0.75 |
|  | min_data_in_leaf | Minimum samples per leaf node | 20 |
|  | lambda_l1 | L1 regularization term | 0 |
|  | lambda_l2 | L2 regularization term | 0 |
| Random Forest | n_estimators | Number of trees | 200 |
|  | max_depth | Maximum depth of the trees | None (no limit) |
|  | min_samples_split | Minimum samples for splitting an internal node | 4 |
|  | min_samples_leaf | Minimum samples per leaf node | 2 |
|  | max_features | Maximum number of features used for splits | sqrt (square root of all features) |
|  | bootstrap | Whether to use sampling with replacement | True |
| Multi-layer perceptron | hidden_layer_sizes | Number of neurons in hidden layers | (50,50) |
|  | activation | Activation function | relu |
|  | solver | Optimization algorithm | adam |
|  | alpha | L2 regularization term | 0.0005 |
|  | learning_rate | Learning rate adjustment method | constant |
|  | max_iter | Maximum number of iterations | 300 |
| Support vector machine | C | Regularization parameter | 1 |
|  | kernel | Type of kernel function | rbf |
|  | gamma | Kernel coefficient | scale |
|  | degree | Degree of polynomial kernel | 4 |
| K-nearest neighbors | n_neighbors | Number of neighbors | 5 |
|  | weights | Weight function for neighbors | uniform |
|  | algorithm | Algorithm for nearest neighbor search | auto |
|  | leaf_size | Size of leaf node | 30 |
|  | p | Power parameter for distance metric | 2 |
| Logistic | penalty | Type of regularization | 10 |
|  | C | Inverse of regularization strength | 1 |
|  | solver | Optimization algorithm | lbfgs |
|  | max_iter | Maximum number of iterations | 150 |
| Gaussian naive bayes | var_smoothing | Smoothing parameter added to the variance | 1e-8 |

**Supplement table 5. B Key Hyperparameters of Machine Learning Algorithms（90 day）**
